# Utilization of the zebrafish model to unravel the harmful effects of biomass burning during Amazonian wildfires

**DOI:** 10.1038/s41598-021-81789-1

**Published:** 2021-01-28

**Authors:** Sanja Babić, Lara Čižmek, Aleksandra Maršavelski, Olga Malev, Maryline Pflieger, Ivančica Strunjak-Perović, Natalija Topić Popović, Rozelindra Čož-Rakovac, Polonca Trebše

**Affiliations:** 1 Laboratory for Aquaculture Biotechnology, Division of Materials Chemistry, Ruđer Bošković Institute, Bijenička 54, Zagreb, Croatia; 2 Center of Excellence for Marine Bioprospecting (BioProCro), Ruđer Bošković Institute, Bijenička 54, Zagreb, Croatia; 3 Faculty of Science, Department of Chemistry, University of Zagreb, Horvatovac 102a, Zagreb, Croatia; 4 Faculty of Science, Department of Biology, University of Zagreb, Roosevelt square 6, Zagreb, Croatia; 5 Laboratory for Biological Diversity, Division for Marine and Environmental Research, Ruđer Bošković Institute, Bijenička 54, Zagreb, Croatia; 6 Faculty of Health Sciences, University of Ljubljana, Zdravstvena pot 5, Ljubljana, Slovenia

**Keywords:** Environmental chemistry, Fire ecology, Embryology, Enzyme mechanisms, Environmental sciences

## Abstract

Amazonian wildfires in 2019 have raised awareness about rainforest burning due to increased emissions of particulate matter and carbon. In the context of these emissions, by-products of lignin thermal degradation (i.e. methoxyphenols) are often neglected. Methoxyphenols entering the atmosphere may form intermediates with currently unknown reaction mechanisms and toxicity. This study for the first time provides a comprehensive insight into the impact of lignin degradation products [guaiacol, catechol], and their nitrated intermediates [4-nitrocatechol, 4,6-dinitroguaiacol, 5-nitroguaiacol] on zebrafish *Danio rerio*. Results revealed 4-nitrocatechol and catechol as the most toxic, followed by 4,6DNG > 5NG > GUA. The whole-organism bioassay integrated with molecular modeling emphasized the potential of methoxyphenols to inhibit tyrosinase, lipoxygenase, and carbonic anhydrase, consequently altering embryonic development (i.e. affected sensorial, skeletal, and physiological parameters, pigmentation formation failure, and non-hatching of larvae). The whole-organism bioassay integrated with in silico approach confirmed the harmful effects of lignin degradation products and their intermediates on aquatic organisms, emphasizing the need for their evaluation within ecotoxicity studies focused on aquatic compartments.

## Introduction

The importance of the Amazonian basin as one of the largest ecosystems on Earth is extensive, from being the richest region hosting 25% of global biodiversity to being a huge carbon sink that eliminates up to 20% of excess atmospheric carbon and contributes to the biogeochemical functioning of the Earth system^1–3^. The Amazon is exposed to different altering factors such as wind blow-downs^4^, droughts^5^, but also deforestation and intensive fires^6^. Only in 2019, Brazil National Institute for Space Research^7^ has recorded 172,214 active fires across Brazil, among which 12,677 took place in the world’s largest rainforest located in the Amazonian basin, popularly known as "Earth's lungs" (Table 1). Although these devastating Amazonian fires ceased, forest burning remains a regular occurrence in the Amazon basin (see current data for the year 2020, Table 1) with an average of 12,817 fires annually.

**Table 1:** The number of total active fires in the Amazonian basin detected by satellite during the last five years^8^.

Year	Month												Total
	1	2	3	4	5	6	7	8	9	10	11	12	
2015	38	71	25	20	11	34	365	*4235*	*5004*	*2233*	909	474	13,419
2016	654	252	105	13	26	84	*1087*	*3652*	*2785*	*1913*	497	105	11,173
2017	62	21	24	15	33	95	*1534*	*4793*	*3185*	*1190*	486	247	11,685
2018	46	93	54	14	19	123	*1346*	*2589*	*4928*	*1725*	472	37	11,446
2019	35	90	114	10	21	57	*1371*	*6669*	*3026*	548	573	163	12,677
2020	197	73	77	12	15	122	*2119*	*8030*	*4270*	*1265*	323^a^	^b^	16,503^a^

Fire occasions that exceeded 1000 are marked in italics.

^a^The latest available data at the time of publication.

^b^Data not available at the time of publication.

The primary concern of long-term vegetation biomass burning and atmosphere perturbation is the concurrent impact on climate and ecosystem function, as well as long-term inhalation of released gases and pollutants which can seriously impact human health, manifesting through respiratory problems, pulmonary disease, nerve disorders, atherosclerosis, or even cancer and death^9,10^. This is also evidenced by the fact that an increase of 59.5% in the number of deaths observed in hospitalized children was reported in Roraima, one of the most fire-affected Amazonian regions in 2019^11^. Particulate matter and carbon are mainly pointed out as sole emissions from these fires^12^, while thermal degradation of lignin by-products (that comprises 18–35% of wood biomass) are often neglected^13^. Pyrolysis of lignin results in the formation of low molecular weight compounds of phenolic structures—methoxyphenols (MPs), which are mainly represented by guaiacol (2-methoxyphenol), catechol (1,2-dihydroxybenzene), and syringol (2,6-dimethoxyphenol)^14,15^. Once MPs reach the atmosphere, they are prone to react with oxidants such as OH and NO_3_ radicals, Cl atom, and ozone molecules^16^. Complex migration and transformation processes of MPs in the troposphere can result in the formation of new airborne pollutants and increase the secondary organic aerosols (SOAs) yield. Although most of the published studies are computational predictions of MPs reactions and their rate constants, these studies are a good indicator of potential degradation products occurring mostly in the gaseous phase yielding from reactions with ozone (primary ozonides—POZ^17^) and NO_3_ (nitro-aromatics derivatives^18^). They can also partition into the aqueous phase and react with OH radicals (phenoxy- and catechol-species)^19^. MPs formed during lignin pyrolysis are found in the atmosphere, ambient particulate matter, drinking and surface water of riverine systems^14,20^. For example, concentration levels of guaiacol in Poland reached up to 0.63 μg/L in a river and 1.37 μg/L in drinking water^14^. Michałowicz^21^ also reported the occurrence of a high concentration of guaiacol exceeding 23 μg/L in Poland’s surface waters. However, information about the occurrence and biological effects of nitrated MPs in aquatic ecosystems are still scarce.

Nitro-aromatic derivates are mostly less volatile and more water-soluble^18^ thus in great proportion remain in the atmospheric aqueous phase. Accordingly, the enrichment of MPs in fog water is usually 3 to 4 times higher than the calculated values, potentially supported by dissolved fog-borne chemicals involved in the solubilization of these compounds^22^. Vast moisture formations in the Amazonian forest (e.g. flying rivers^7^) rise via convection and collide with the stratosphere enabling a majority of chemical compounds, including nitrated GUA and CAT intermediates, to easily cross the moisture column and reach the Earth's surface^23^. Thus, guaiacol, syringol, and their nitrated or chlorinated MPs can accumulate in different environmental compartments and food chains exerting potential toxic effects on non-target organisms including humans.

Despite a well-known fact that nitro groups in the molecular structure increase toxicity of aromatic phenols, to date there are no comprehensive data on the amount of guaiacol and catechol nitrated forms that could affect vertebrates, and ultimately humans. To our knowledge, Pflieger and Kroflič^24^ are the first that provided toxicological data for guaiacol and its nitro derivatives using marine bioluminescent bacterium *Vibrio fischeri* as a model test organism. Although this study revealed the harmful potential of nitrated guaiacol and pointed out the need for further toxicological testing, currently there are no other studies that address this class of chemicals in detail.

To fill this knowledge gap, our study focused on zebrafish *Danio rerio* as an in vivo vertebrate model platform which enabled high-throughput screening and (eco)toxicity evaluation of two common MPs formed during lignin pyrolysis—guaiacol (GUA) and catechol (CAT), and their nitrated forms: 4,6-dinitroguaiacol (4,6DNG), 5-nitroguaiacol (5NG) and 4-nitrocatechol (4NC). The whole-organism bioassay was integrated with in silico methods based on: (i) quantitative structure–activity relationship (QSAR) computation model for comparison of predicted lethal concentrations to fish with determined toxicity values, and (ii) molecular modeling focused on interactions of tested compounds with zebrafish main protein targets. MPs and nitro-MPs toxicity can arise from a variety of reasons: specific highly-reactive functional groups, the compounds’s physicochemical properties, and the ability of the compound to bind to specific protein targets. As toxic potential of MPs can be caused by various parameters, a comprehensive approach utilizing three complementary types of data (i.e. in silico and in vivo data) applied in our study may improve the prediction and attempt to clarify MPs (or nitro-MPs) toxicity at organism level.

## Materials and methods

### Chemicals

Guaiacol (HPLC/GC grade; CAS No. 90-05-1; GUA), 5-nitroguaiacol (98%; CAS No. 636-93-1; 5NG), catechol (≥ 95.0%; CAS No. 120-80-9; CAT), 4-nitrocatechol (97%; CAS No. 3316-09-4; 4NC), ethyl 3-aminobenzoate methanesulfonate salt (HPLC/GC grade; CAS No. 886-86-2; MS-222) were purchased from Sigma-Aldrich (Deisenhofen, Germany). 4,6-dinitroguaiacol (95%; CAS No. 19978-25-7; 4,6DNG) was obtained from Debye Scientific Co, Ltd. (Hong Kong, China). Artificial water^25^ was prepared using chemicals all purchased from Sigma Aldrich (Deisenhofen, Germany): calcium chloride dihydrate (≥ 99%; CAS No. 10035-04-8; CaCl_2_ × 2H_2_O), magnesium sulfate heptahydrate (≥ 98%; CAS No. 10034-99-8; MgSO_4_ × 7H_2_O), sodium bicarbonate (≥ 99.7%; CAS No. 144-55-8; NaHCO_3_), potassium chloride (≥ 99%; CAS No. 7447-40-7; KCl).

### Ethics statement

Animal housing and spawning were performed in aquaria units approved by the Croatian Ministry of Agriculture and according to the Directive^26^. All experiments in this study were conducted on the non-protected embryonic stages (up to 96 hpf), which do not require permission by animal welfare commissions^26^.

### Zebrafish maintenance and embryo production

Zebrafish *D. rerio* [wildtype WIK strain obtained from the European Zebrafish Resource Center of the Karlsruhe Institute of Technology (KIT), Germany] were maintained in ZebTEC rack with Active Blue technology (Tecniplast S.p.A., Buguggiate, Italy) under a continuous photoperiod cycle of 14:10 h (light: dark). The water parameters were strictly controlled: temperature of 27.00 ± 0.09 °C, conductivity at 494.80 ± 2.61 µS/cm, pH at 7.60 ± 0.08, dissolved oxygen ≥ 95% saturation. Adults were fed three times per day with frozen *Artemia* sp. (PETRA-AQUA tropical fish wholesale, Czech Republic).

A day before the experiment, males and females were transferred into the iSpawn-S Benchtop Size Breeding System (Tecniplast S.p.A.) at a 2:1 male to female ratio and kept separated by a divider. The next day, the divider was removed, and the spawning platform was lifted to initiate the spawning. After spawning eggs were collected within 15 min using an 800 μm mesh and were rinsed to remove the debris.

### Zebrafish embryotoxicity test (ZET)

The test was performed according to the OECD Test Guideline^27^, with slight modifications. During dose range-finding experiments, embryos (n = 10 per concentration) were exposed to a wide range of concentrations spanning from 1.17 up to 300.00 mg/L which were prepared in serial dilutions to obtain a testing range of interest, after which the main experiment was conducted.

Then, the main experiment was conducted in order to determine EC_50_ (half maximal effective concentration) and LC_50_ (half maximal lethal concentration) values. Fertilized eggs from 4- to 64-blastomeres were selected under a stereomicroscope (PRO-LUX, Croatia) and transferred individually into 24-well plates (NEST Scientific, USA) containing a final volume of tested sample (2 mL per well). Plates were incubated at 27.00 ± 0.5 °C in the Innova 42 incubator shaker (New Brunswick, Canada). Due to the photosensitivity of tested compounds, during the whole experiment specimens were kept in the dark. Daily, 30% of the test sample was replaced with a previously pre-warmed, aerated, and freshly prepared test solution^27^. For incubation and dilution of test solutions, artificial water^25^ (294.0 mg/L CaCl_2_ × 2 H_2_O; 123.3 mg/L MgSO_4_ × 7 H_2_O; 63.0 mg/L NaHCO_3_; 5.5 mg/L KCl) was used. Negative controls within the experiment consisted of both internal (n = 4 embryos for plate control) and external (n = 24 embryos on additional plate) controls. In all test solutions, pH was adjusted to 7.40 ± 0.21. The test was conducted with 10 embryos in three independent replicates, amounting to a total of 30 embryos per concentration. Every day lethal and sub-lethal effects were recorded using an inverted microscope (Olympus CKX41), equipped with Leica EC3 digital camera and LAS EZ 3.2.0 digitizing software (https://www.leica-microsystems.com/products/microscope-software/p/leica-las-ez/). At 96-h post-fertilization (hpf) percent of hatched larvae was also observed.

### Morphometric analysis

After 96 h of exposure, 15 zebrafish larvae were randomly selected from each group and anesthetized with 100 mg/L of MS-222. Morphometric analysis was conducted on zebrafish larvae exposed to the corresponding LC_50_ value of each compound: 10.95 mg/L of CAT, 8.16 mg/L of 4NC, 211.40 mg/L of GUA, 76.02 mg/L of 5NG, and 25.82 mg/L of 4,6DNG. Such high concentrations (LC_50_ values) were chosen to determine which parameter, whether sensorial (eye area), physiological (yolk and pericardial sac area), or skeletal (head height) was the most affected under increased chemical load. The analysis was conducted using the Olympus BX51 light binocular microscope, while the measurements were performed using Microsoft AnalySIS Soft Imaging System software for DP70 Camera (https://www.olympus-lifescience.com/en/support/downloads/).

### Computational methods

The model of zebrafish carbonic anhydrase (CA; UniProt entry: Q92051) was modeled by I-TASSER server^28^. Only one model of zebrafish CA was obtained with C-score of 1.7 out of 2.0, which accounts for a highly reliable model. Structure templates identified in the Protein Data Bank (PDB) library are 3mdz, 1fql, 3kwa, 1czm, 3uyq, and 1flj. These PDB entries correspond to different human (3mdz, 1fql, 3kwa, 1czm, 3uyq) and rat (1flj) CA isozymes. The model structure of zebrafish CA obtained by I-TASSER server is deposited in ModelArchive (Project ID: ma-j4w5e). The homology model is validated by SAVES v5.0 server which showed good statistics for stereochemical, non-bonded interactions, and geometric parameters. Coordinates of Zn cation and water molecule coordinated to Zn cation were taken from the structure of carbon anhydrase deposited under PDB ID 1fql. Structures of ligands (chemical structures of tested compounds) were taken from ZINC database^29^: guaiacol, GUA (ZINC13512224); 5-nitroguaiacol, 5NG (ZINC00295034), 4,6-dinitroguaiacol, 4,6DNG (ZINC4343573), catechol, CAT (ZINC13512214) and 4-nitrocatechol, 4NC (ZINC34800312). In this study, we used SwissDock server and AutoDock Vina 1.1.2 to dock the above-mentioned ligands to modeled CA^30^. The default parameters were used and the box centre in the receptor coordinate system was calculated by Ghecom 1.0^31^ and set to 66.181 53.296 69.746 whereas box dimensions were set to 20, 20, 20 Å. This corresponds to the active site that contains Zn cation coordinated to three histidine moieties. Obtained docking results were analyzed in Chimera^32^.

### Statistical analysis

**Figure 1:**
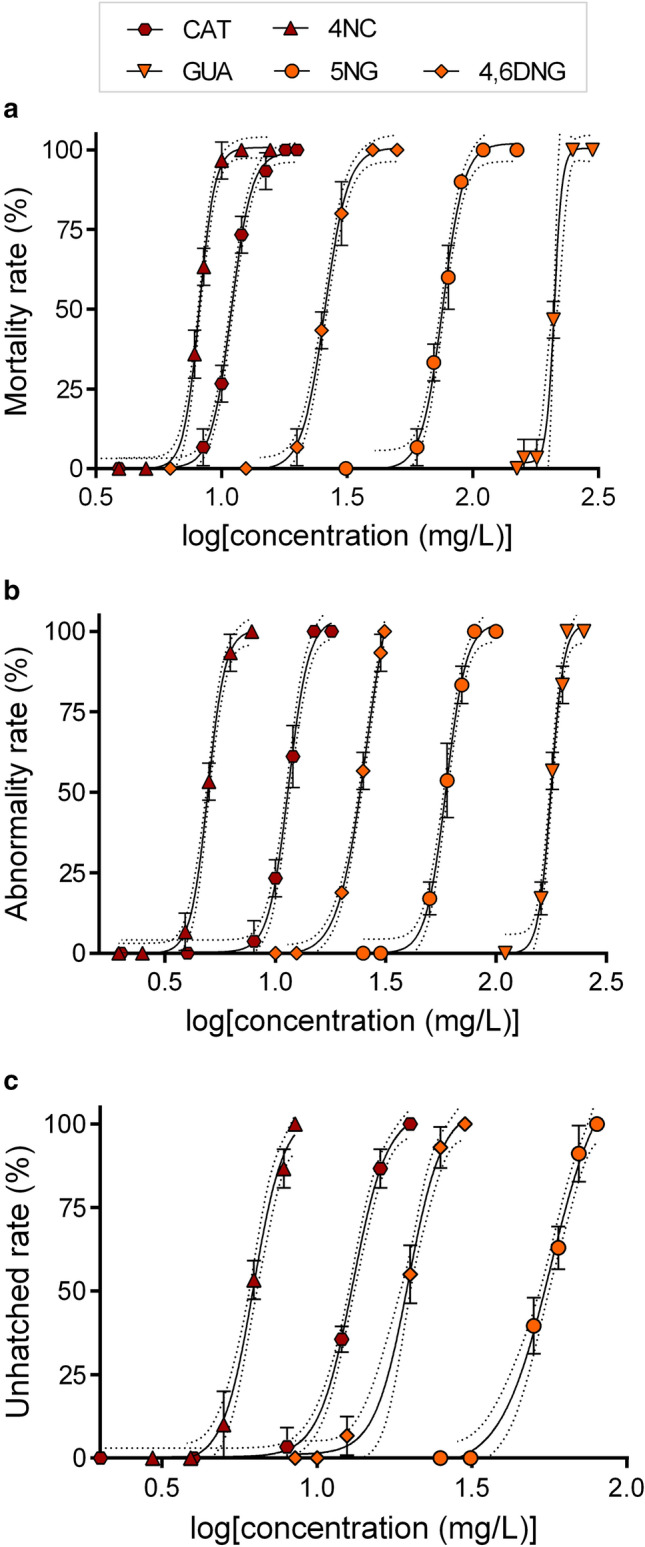
Concentration–response curves used for the calculations of the *D. rerio:* (**a**) mortality, (**b**) abnormality, and (**c**) unhatching rate after 96 h of exposure to CAT, GUA, 4NC, 5NG, and 4,6DNG. Error bars indicate standard deviations (SD). Dotted lines represent 95% confidence intervals. Dose–response curves were generated using GraphPad Prism software version 6.0 (https://graphpad-prism.software.informer.com/6.0/).

Statistical analysis and graphical representation were performed using GraphPad Prism software version 6.0 (https://graphpad-prism.software.informer.com/6.0/). Prior to LC_50_/EC_50_ determination, data were subjected to logarithmic transformation (Fig. 1).

One-way analysis of variance (ANOVA) and Tukey’s post hoc test were performed to examine the significance between negative control and tested samples, as well as among treatments. When the assumption for normality was violated the Kruskal–Wallis One-way analysis of variance on ranks was performed. The results were expressed as means ± SD, and p ≤ 0.05 was used as a cut-off value of statistical significance throughout the paper.

Dark-red colour represents values and endpoints observed for CAT and 4NC, while orange colour represents GUA and its nitrated intermediates (5NG, 4,6DNG) in Figs. 1 and 4, as well as in Table S1.

## Results and discussion

### In vivo study: embryotoxicity test

**Figure 2:**
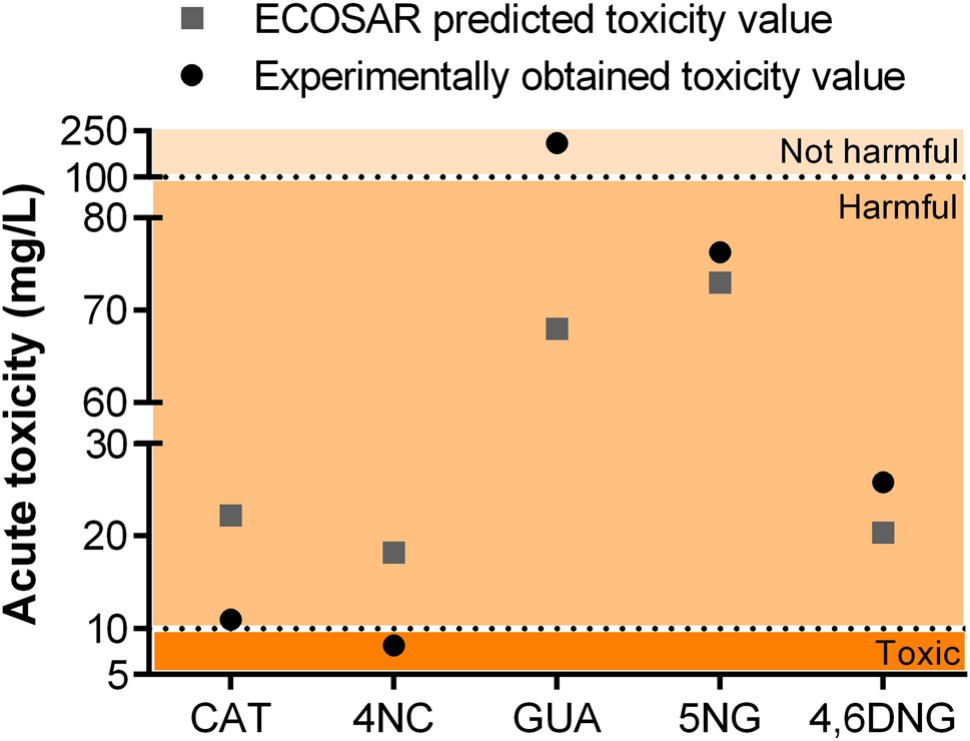
Graphical view of ECOSAR predicted and experimentally obtained toxicity values within the toxicity gradient according to the criteria set by the European Union^34^ (Very toxic < 1 mg/L, Toxic 1 < LC_50_ < 10 mg/L, Harmful 10 < LC_50_ < 100 mg/L, Not harmful LC_50_ > 100 mg/L). Predicted values were obtained from ECOSAR v2.0 software (https://www.epa.gov/tsca-screening-tools/ecological-structure-activity-relationships-ecosar-predictive-model), while GraphPad Prism software version 6.0 (https://graphpad-prism.software.informer.com/6.0/) was used for data presentation.

Zebrafish embryos exposed to tested compounds developed lethal and sub-lethal alterations including different abnormalities and unhatching events. LC_50_ (for mortality rate) and EC_50_ (for abnormality and unhatching rate) values were extrapolated from concentration–response curves shown in Fig. 1. The rate of dead, abnormal, and/or unhatched specimens was concentration-dependent for all tested compounds (Fig. 1a–c). The lethality of the negative control group was less than 5%. Compounds 4NC and CAT showed the highest toxicity with LC_50_ values of 8.16 and 10.95 mg/L, respectively, followed by 4,6DNG > 5NG > GUA. Experimental LC_50_/EC_50_ values and the predicted ones obtained by ECOlogical Structure Activity Relationship (ECOSAR) v2.0 software (https://www.epa.gov/tsca-screening-tools/ecological-structure-activity-relationships-ecosar-predictive-model) based on Quantitative Structure Activity Relationships (QSAR) models showed 4NC and CAT as the most toxic chemicals (Table 2). However, it is important to notice that experimental values for both compounds were approximately two times lower than the predicted ones. This led to the classification of 4NC into the group of molecules toxic to fish (1 < LC_50_ < 10 mg/L), and not only harmful as predicted by ECOSAR (Fig. 2). Furthermore, our study showed that GUA, although considered as harmful to fish, classifies as not harmful to zebrafish. As a result, estimated ECOSAR values could not be used for quantitative environmental risk assessment nor for prediction on compound's toxicity that could be applied for all fish species. In this study, ECOSAR-predicted values were provided for comparison to the obtained experimental values only. Differences between predicted and experimental values underline the increasing importance of accompanying in silico methods with in vivo toxicological tests, which is the only way to determine the compound's realistic toxic potential and evaluate its impact on the aquatic ecosystem. These issues are presented schematically in Fig. 2.

**Table 2:** Acute toxicity of guaiacol, catechol, and its nitrated products (mg/L): tabular view of toxicological predictions obtained from ECOSAR software for 96-h exposed fish and obtained experimental values for 96-h exposed zebrafish *D. rerio* embryos.

(a)	Tested compounds	Experimental data	Ecotoxicological predictions
		LC_50_ 96-h (mg/L)	LC_50_ 96-h fish (mg/L)
Mortality	Catechol (CAT)	10.95 ± 0.21	22.23
	4-Nitrocatechol (4NC)	8.16 ± 0.10	18.21
	Guaiacol (GUA)	211.40 ± 2.2	68.73
	5-Nitroguaiacol (5NG)	75.96 ± 1.92	46.87
	4,6-Dinitroguaiacol (4,6DNG)	25.82 ± 0.64	20.39

(b)	Tested compounds	EC_50_ 96-h (mg/L)	
Developmental abnormalities	Catechol (CAT)	11.49 ± 0.32	
	4-Nitrocatechol (4NC)	4.95 ± 0.10	
	Guaiacol (GUA)	177.80 ± 3.30	
	5-Nitroguaiacol (5NG)	59.42 ± 1.64	
	4,6-Dinitroguaiacol (4,6DNG)	25.80 ± 1.74	
Unhatching	Catechol (CAT)	13.03 ± 0.43	
	Guaiacol (GUA)	> 300*	
	4-Nitrocatechol (4NC)	6.23 ± 0.29	
	5-Nitroguaiacol (5NG)	56.80 ± 5.39	
	4,6-Dinitroguaiacol (4,6DNG)	19.70 ± 0.76	

Predicted values were obtained from ECOSAR v2.0 software (https://www.epa.gov/tsca-screening-tools/ecological-structure-activity-relationships-ecosar-predictive-model).

*Maximal effect not reached.

Differences in toxicity between CAT, GUA, and nitrated intermediates were already observed elsewhere^24^ and are highly dependent on the position of nitro groups on the benzene ring^33^. In their study, Pflieger and Kroflič^24^ observed an inhibitory effect on *V. fischeri* luminescence, which was 6-folds higher for bacteria exposed to 4,6DNG (EC_50_ = 16.7 mg/L) than the ones exposed to GUA (100.6 mg/L). A similar, although more pronounced toxicity trend was observed within this work, showing an eightfold higher toxicity of 4,6DNG compared to its non-nitrated form (Fig. 1a; Table 2). Such findings confirm the high sensitivity of zebrafish embryos to nitrated MPs and their toxicity potential due to their specific highly-reactive functional groups and chemical properties. Furthermore, as ECOSAR uses only the partitioning coefficient (log P) to predict fish toxicity it could underestimate MPs and nitro-MPs real toxic potential for chemicals acting through specific interactions and non-covalent binding to enzymes. This potential mode of action of MPs is also addressed in “In silico study: molecular modeling”.

**Figure 3:**
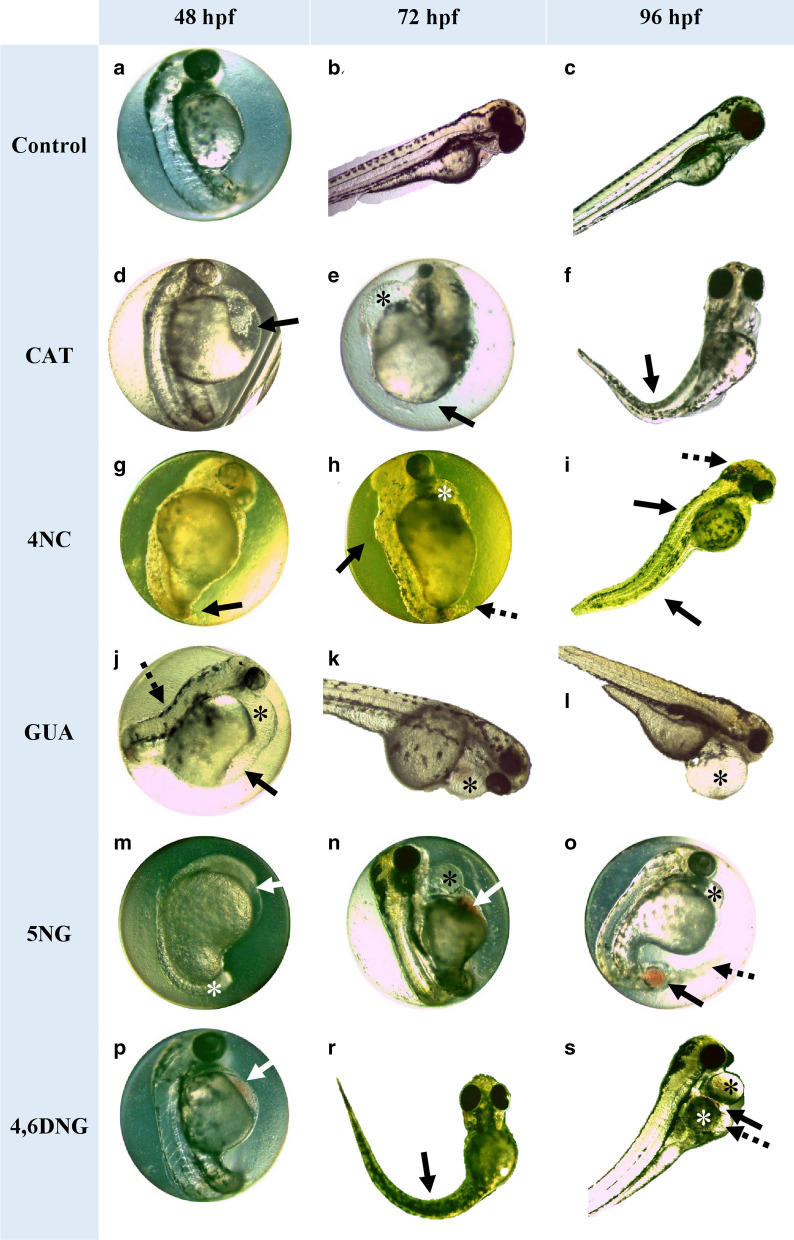
Recorded sublethal morphological effects in *D. rerio* embryos/larvae after 48, 72, and 96 h of exposure to CAT, 4NC, GUA, 5NG, and 4,6DNG. Negative control: normally developed embryo at (**a**) 48, (**b**) 72, and (**c**) 96 hpf. During exposure period alterations were manifested as: (**d**) yolk sac edema (arrow); (**e**) pericardial edema (asterisk), undeveloped tail region (arrow); (**f**) hatched fish with malformed spine (arrow); (**g**) underdeveloped tail and necrosis of its apical part (dashed arrow), rare pigments; (**h**) pericardial edema (asterisk), scoliosis (arrow), necrosis of the apical part of the tail (dashed arrow), rare pigments, not hatched; (**i**) scoliosis (arrows), blood accumulation in the brain region (dashed arrow); (**j**) pericardial edema (asterisk), yolk sac edema (arrow), scoliosis (dashed arrow); (**k**, **l**) pericardial edema (asterisk); (**m**) underdeveloped embryo: underdeveloped head (arrow), tail not detached (asterisk), delay or anomaly in the absorption of the yolk sac; (**n**) pericardial edema (asterisk), blood accumulation (arrow), not hatched; (**o**) pericardial edema (asterisk), blood clotting (arrow), not hatched; (**p**) blood accumulation at the yolk sac (arrow); (**r**) hatched fish with malformed spine; (**s**) pericardial edema (black asterisk), blood accumulation above the yolk sac (arrow), swelling of the yolk sac (white asterisk), yolk sac edema (dashed arrow), mild scoliosis. Developmental abnormalities were recorded using LAS EZ 3.2.0 digitizing software (https://www.leica-microsystems.com/products/microscope-software/p/leica-las-ez/).

The most dominant abnormalities observed during the exposure to tested compounds were pericardial edema (Fig. 3e,h,j–l,n,o,s), blood accumulation in the yolk sac (Fig. 3n,p,s), and at later developmental stages (72, 96 hpf) skeletal deformities (Fig. 3f,h,i,r,s), undeveloped tail and necrosis of its apical part (Fig. 3g,h) and yolk sac edema (Fig. 3d,j,s). During the exposure to nitrated intermediates, blood accumulation in the brain region (Table S1, Fig. 3i) was also observed. Besides developmental abnormalities, the two most commonly observed endpoints were pigment formation failure (Fig. 3g,m) and non-hatching of larvae (Fig. 3e,h,n,o). Control group on artificial water developed normally (Fig. 3a–c). Based on Fig. 3 and Table S1 it can be concluded that the type of abnormality was not compound-related, but the sum of all obtained developmental abnormality types recorded on tested compounds (4NC > CAT = 4,6DNG = 5NG > GUA) mostly follows their toxic potential trend (4NC > CAT > 4,6DNG > 5NG > GUA).

**Figure 4:**
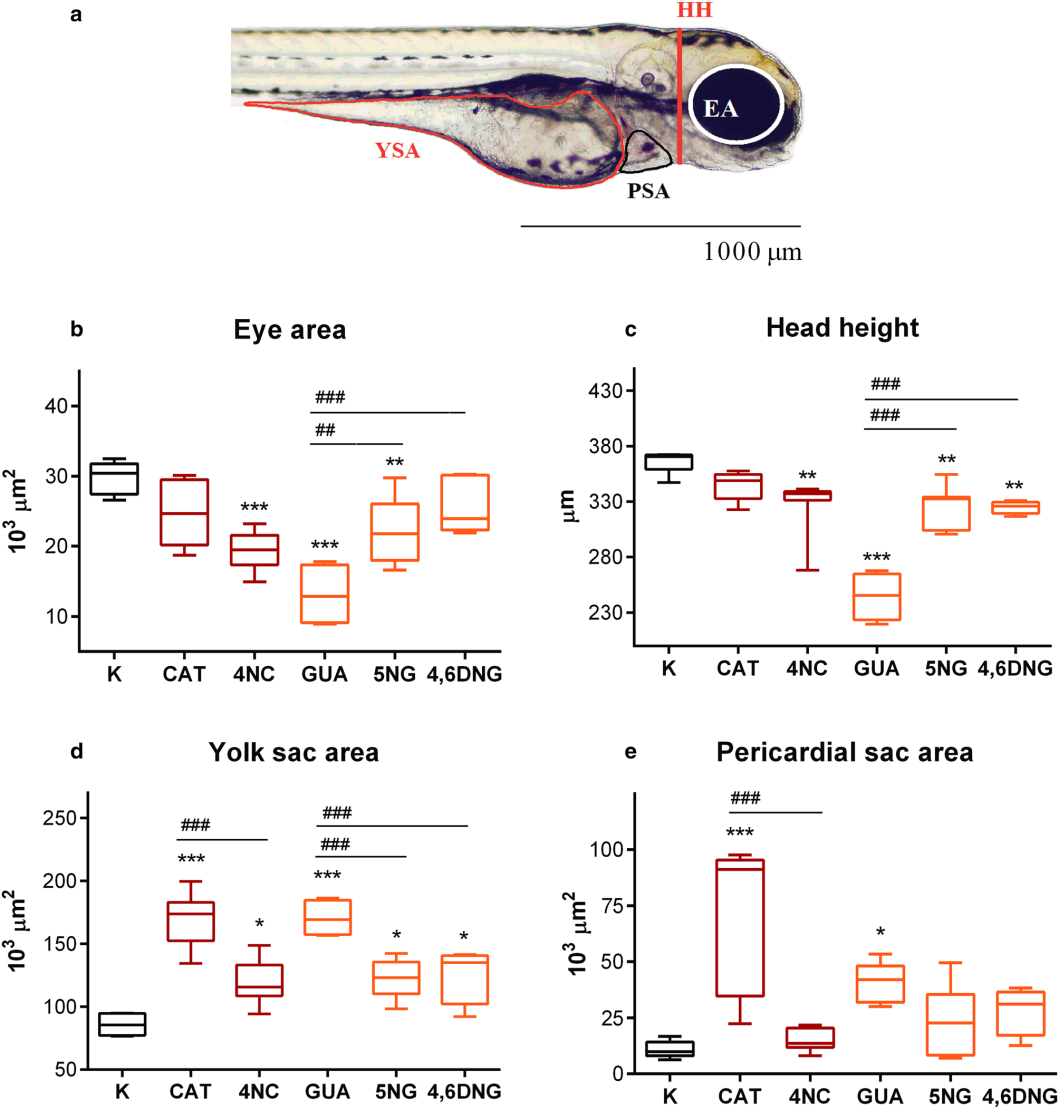
Morphometric measurements of *D. rerio* larvae after 96-h exposure to tested compounds (CAT, 4NC, GUA, 5NG, and 4,6DNG) and control (C). (**a**) Lateral view showing eye area (EA), head height (HH), yolk sac area (YSA), and pericardial sac area (PSA). Scale bar = 1000 µm. Morphometric parameters are presented by their mean value (**b**–**e**; n = 15). The symbol * indicates a significant difference between tested samples and negative control (*p < 0.05; **p < 0.01; ***p < 0.001). Mean values sharing common letters indicate significant differences among different tested compounds (*p < 0.05). A line within the box represents the median value, while the boundaries of box-plot indicate 25th and 75th percentiles. Whiskers above and below the box indicate 10th and 90th percentiles. Statistical analysis and data presentation were prepared using GraphPad Prism software version 6.0 (https://graphpad-prism.software.informer.com/6.0/), while visualization and morphometric measurements were performed using Microsoft AnalySIS Soft Imaging System software (https://www.olympus-lifescience.com/en/support/downloads/).

The morphometric measurements (Fig. 4) showed that all tested samples significantly affected sensorial (eye area), skeletal (head height), and physiological (yolk and pericardial sac area) parameters in zebrafish. Significant differences among all treatments with exact p values are presented in Table S2.

The highest impact was noted on the size of the yolk sac area (Fig. 4c). A statistically significant increase of yolk sac area was observed among all tested samples, thus indicating a potential decline in metabolic rate and consequent decline in yolk consumption. The highest values of the yolk sac area were noted in larvae exposed to CAT (169.1 × 10^3^ µm^2^) and GUA (170.3 × 10^3^ µm^2^) compared to the control group (85.9 × 10^3^ µm^2^). It is important to emphasize that yolk consumption delay was not caused by growth retardation. One of the most frequently recorded sublethal endpoints during CAT and GUA exposure was pericardial edema (Table S1) which resulted in pericardial sac enlargement (169.1 and 170.3 × 10^3^ µm^2^, respectively, compared to the control value of 85.9 × 10^3^ µm^2^) (Fig. 4d). CAT and GUA significantly impacted both measured physiological parameters, while skeletal structural parameters (Fig. 4a, b) were severely affected by GUA and nitrated intermediates. Compared to the control of untreated specimens, GUA caused the highest decrease in the zebrafish eye area (13.1 × 10^3^ µm^2^ compared to the control values of 29.9 × 10^3^ µm^2^) and head height (244.8 µm compared to the control values of 366.1 µm). The same decline in skeletal parameter values was noted during exposure to 4NC and 5NG, and 4,6DNG (10.1–11.3% decrease compared to the control values). This type of morphological measurement could serve as a valuable additional tool that has the potential to mitigate errors and limitations of qualitative analysis.

### In silico study: molecular modeling

A recent study has shown that GUA and CAT effectively inhibit different human CA isoenzymes with *K*_i_ in mmol/L range^35^. CA is a zinc-containing enzyme that catalyzes reversible interconversion between carbon dioxide and water into protons and bicarbonate ions. During zebrafish early development (24–48 hpf), embryos use CA for increased CO_2_ excretion^36^. The activity of zebrafish CA5 isozyme is shown to be essential for the regulation of acid–base balance during embryonic growth and its inhibition causes developmental abnormalities during embryonic development^37^. It was already shown that phenol and 2-nitrophenol are inhibitors of a cytosolic, human CA isoform II (hCA II)^38,39^. Namely, phenols bind to zinc-bound water through their OH moiety whereas the phenyl moiety is located in the hydrophobic part of the hCA II active site preventing binding of substrate CO_2_, thereby acting as inhibitors^40^.

**Figure 5:**
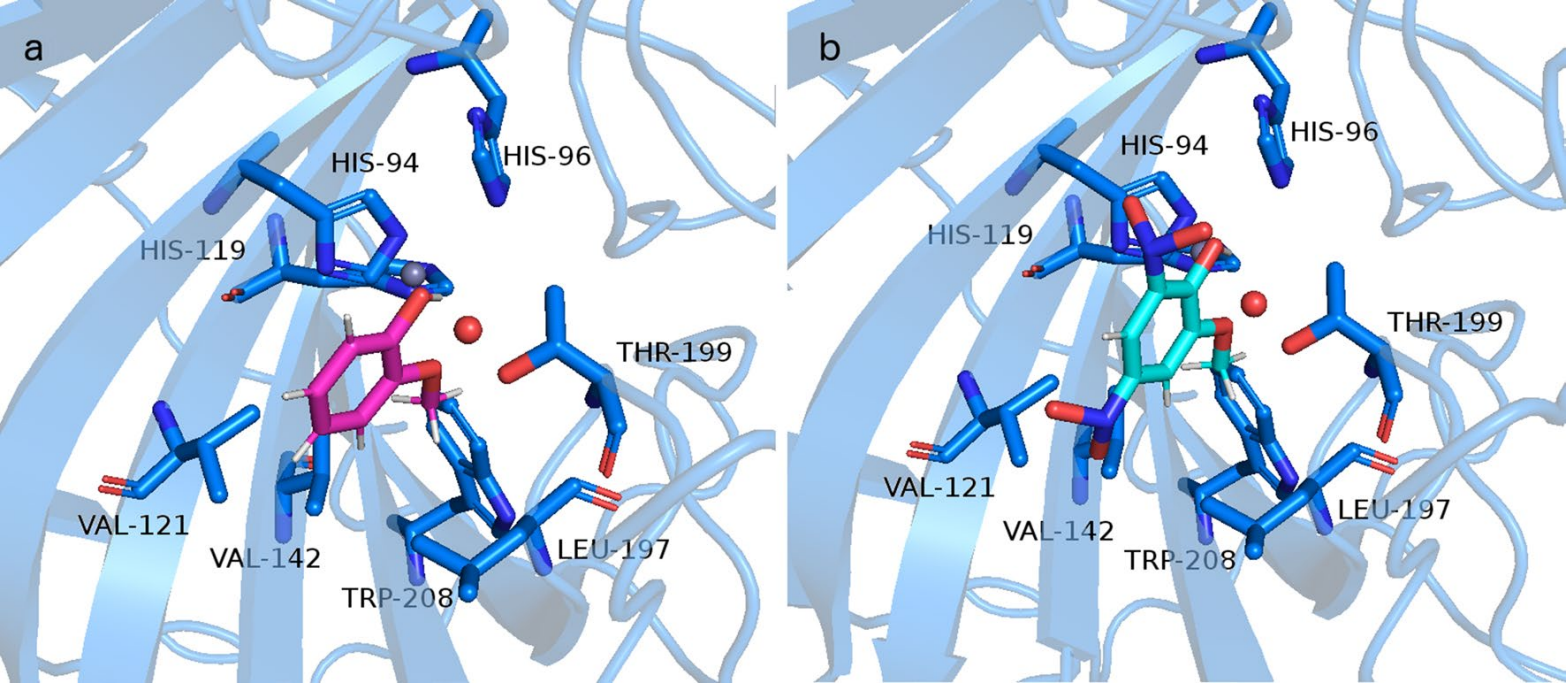
Representation of the interactions between (**a**) GUA and (**b**) 4,6DNG with the active site *D. rerio* CA II. Structure of *D. rerio* CA II (UniProt entry: Q92051) is modeled by I-TASSER, coordinates for Zn cation, and active site water molecule (oxygen atom in red) were taken from the structure of human CA deposited under PDB ID 1fql, whereas docking pose for GUA was obtained by SwissDock server. The same pose was obtained by AutoDock vina. Representation was made in PyMOL software (https://pymol.org/2/).

Uniprot database possesses one manually annotated structure of zebrafish CA (CAH-Z), EC 4.2.1.1. (UniProt ID Q92051) that consists of 260 amino acids. Titration of this zebrafish CA isozyme with ethoxzolamide (EZA), a sulfonamide inhibitor, resulted in the subnanomolar *K*_i_ which was an indication that this zebrafish CA is a homolog to hCA II, which is also inhibited by sulfonamide inhibitors^41^. Alignment of zebrafish CA (CAH-Z) and hCA II, whose structure has been solved, resulted in 63% identities and 76% positives in primary sequences that account for highly homologous enzymes (Fig. S1). In addition to sulfonamide inhibitors, the previous study showed that catechols also inhibit hCA II isoform and because of high conservation in primary sequence between hCA II isoform and zebrafish CA isozyme we considered that catechol also inhibits zebrafish CA^42^. Therefore, we have modeled a 3D structure of zebrafish CA by using the I-TASSER server. With the obtained model we performed docking of the compounds tested in this study—CAT, GUA, 4NC, 5NG, and 4,6DNG. Docking proposed the same mode of binding as was previously explained for phenol, CAT, and GUA intermediates^35,40^. The corresponding predicted binding free energies of the docked structures (CAT, GUA, 4NC, 5NG, and 4,6DNG) obtained by AutoDock Vina are − 4.4, − 4.5, − 5.3, − 5.5, − 5.9 kcal/mol and by SwissDock are − 6.2, − 6.1, − 6.3, − 6.2, − 6.6 kcal/mol, respectively. Lignin pyrolysis products and their nitrated intermediates bind within the *D. rerio* CA binding site in the same manner as was described for hCA II. Namely, in the case of GUA, interactions are made between GUA –OH moiety and water bound to the Zn cation. Methoxy moiety of GUA makes interactions with side-chain of Thr199, which is conserved amino acid among all CA, whereas methyl group from methoxy moiety and phenol part of the GUA make hydrophobic interactions with Leu197, Trp208, Val121, and Val142 (Fig. 5a). According to the docking results, all docked compounds have comparable binding free energies (within the standard error of the scoring function) to 4,6DNG showing slightly better affinities towards *D. rerio* CA. The obtained pose from 4,6DNG is virtually the same as described for GUA (Fig. 5b). It is known that nitro compounds (−NO_2_) have a positive electrostatic potential named π-hole which can establish favorable interactions with lone-pair electrons^43^. Nitro group at the 6-position is directed towards the lone pairs of Ser56 −OH of the side chain, and side chains of Asn62 and Gln67 making favorable interactions.

In addition to inhibition of CA, recent studies have shown that our tested compounds can inhibit other enzymes shown to be essential for zebrafish embryo development^44–47^. Embryotoxicity results obtained within this study indicated that 4NC, followed by CAT, GUA, 4,6DNG, and 5NG notably reduce or even prevent the formation of melanin (Table S1). Such a reduction/absence of pigmentation is usually caused by the ability of a compound to inhibit copper-containing enzyme tyrosinase, thus preventing the conversion of tyrosine into melanin^48^. CAT, GUA, and nitrated intermediates can serve as alternative substrates for tyrosinase, competing with tyrosine, which in turn results in reduced or even inhibited synthesis of melanin^44–47^. For instance, it has been already observed that tyrosinase from *S. glaucescens* is strongly inhibited by 4NC^49^. Therefore, the delay and absence of pigmentation observed within this study is most certainly the result of tyrosinase inhibition due to specific interactions with tested compounds (Table 2).

It has also been shown that 4NC strongly inhibits the iron-containing enzyme lipoxygenase that catalyzes the oxidation of unsaturated fatty acids to yield fatty acid hydroperoxides^50^. When the gene for lipoxygenase is subjected to the targeted knock-down, the zebrafish embryo displays an abnormal phenotype characterized by malformation of the brain, the eyes, and the tail as well as pericardial and yolk sac edema^51^. Therefore, compounds that inhibit lipoxygenase lead to the above-mentioned severe phenotype, which was also observed within this study.

GUA, CAT, and their nitrated derivatives 4NC, 4,6DNG, and 5NG act jointly on different enzymes as targets (i.e. tyrosinase, lipoxygenase, and CA), leading to the abnormal phenotype that has been observed. Previous studies have shown that these enzymes are indeed important for the normal development of zebrafish specimens, whereas their inhibition or decreased expression leads to concurrent abnormalities that have been reported.

Our study has pinpointed 4NC and CAT as the most toxic compounds in the embryotoxicity test followed by 4,6DNG, 5NG, GUA, respectively. On the other hand, our docking study has revealed that all tested compounds in interaction with *D. rerio* CA II have binding free energies within the standard error of the scoring function making those compounds equally good inhibitors of this enzyme. Thus, tested compounds most likely act jointly on many different enzymes resulting in the abnormal phenotype that has been observed within our experiments.

## Conclusion

Wood fires are the main part of an ongoing deforestation trend that reached the most concerning levels during an Amazonian biggest wildfire in 2019, as well as fires in Australia in 2019 and 2020. Amazonian wildfire in 2019 caused the deforestation of almost one million hectares and produced 140 million metric tons of carbon dioxide. Considering that softwood and hardwood burning release up to 0.36 and 0.19 mg of guaiacol, 7.11 and 5.43 mg of catechol, and 0.26 and 15.35 mg of syringol, respectively, per g of emitted organic carbon, we can assume that several hundred tons of various MPs were released into the atmosphere in 2019 during these burning events. Such continuous input of MPs and their nitrated compounds into the aquatic environment could lead to currently unknown consequences if introduced in the food chain and river sediment.

Data reported in this study showed CAT and its nitrated intermediate (4NC) as the most toxic compounds, followed by 4,6DNG, 5NG, and GUA. The harmful effect of MPs on zebrafish embryonic development was manifested on multiple levels including cellular (i.e. inhibition of tyrosinase, lipoxygenase, and CA activity), morphological (i.e. head length and eye area reduction), physiological (i.e. delay in yolk consumption), and at the whole organism (i.e. occurrence of developmental abnormalities) levels. One of the first effects that occurred in zebrafish were developmental abnormalities and delayed hatching that consequently disturbed normal development and increased vulnerability to predation. Overall, observations recorded during MPs and nitro-MPs exposure demonstrated that zebrafish sensitivity can aid in early-pollution monitoring of these compounds in aquatic ecosystems.

Our data give an initial insight into the MPs toxicity potential to aquatic organisms (i.e. fish) and in particular to their early embryonal development. However, several limitations should be acknowledged. First, although the zebrafish embryotoxicity test and obtained results indicate significant progress and represent a reliable and inexpensive platform for further MPs testing, the system requires further expansion with other model aquatic organisms that might express different sensitivity levels. Furthermore, complex chemical processes and additional known/unknown product formation which occur during lignin thermal degradation and atmospheric reactions should be evaluated performing studies under real environmental conditions and taking into account potential synergistic actions. The limitation of QSAR predictive outputs could be overcome (but not completely) by integrating multiple computational models with different predictors, which is among all other mentioned issues a scope of our further studies.

Overall, only continuous research on atmospheric reactions, and identification of new intermediates in the environment, along with toxicological screening, will provide new data about fire-emitted products and their potential impact on our environment and health. Considering the increase of wildfire occurrences and their future trend under climate change, we believe that this study will serve as a base for all future investigations on MPs.

## Supplementary Information

Supplementary Information.
