# Supplementary material for: Utilization of the zebrafish model to unravel the harmful effects of biomass burning during Amazonian wildfires
Source: Sci Rep. 2021 Jan 28;11:2527. doi: 10.1038/s41598-021-81789-1 (PMC7844006; doi:10.1038/s41598-021-81789-1)
Supplement: Supplementary file 1 — Supplementary Information. [file 41598_2021_81789_MOESM1_ESM.pdf]

Supplementary material

Utilization of the zebrafish model to unravel the harmful effects of biomass burning during Amazonian wildfires

Sanja Babić[1,2], Lara Čižmek[1,2], Aleksandra Maršavelski[3], Olga Malev[4,5]*, Maryline Pflieger[6], Ivančica Strunjak-Perović[1,2], Natalija Topić Popović[1,2], Rozelindra Čož-Rakovac[1,2], Polonca Trebše[6,*]

[1]Ruđer Bošković Institute, Laboratory for Biotechnology in Aquaculture, Division of Materials Chemistry, Bijenička 54, Zagreb, Croatia

[2]Ruđer Bošković Institute, Center of Excellence for Marine Bioprospecting (BioProCro), Bijenička 54, Zagreb, Croatia

[3]University of Zagreb, Faculty of Science, Department of Chemistry, Horvatovac 102a, Zagreb, Croatia

[4]University of Zagreb, Faculty of Science, Department of Biology, Roosevelt square 6, Zagreb, Croatia

[5]Ruđer Bošković Institute, Laboratory for Biological Diversity Division for Marine and Environmental Research, Bijenička 54, Zagreb, Croatia

[6]University of Ljubljana, Faculty of Health Sciences, Zdravstvena pot 5, Ljubljana, Slovenia

Correspondence to Polonca Trebše (polonca.trebse@zf.uni-lj.si) and Olga Malev (olga.malev@irb.hr)

Table S1. Developmental abnormalities observed in zebrafish *D. rerio* (n=30) during 96 hours of exposure to CAT and GUA, and their nitrated intermediates. Often, several developmental abnormalities were observed for a single zebrafish.

**Toxicological endpoint**	Observation time (hpf)				**CAT**	**4NC**	**GUA**	**5NG**	**4,6DNG**
	24	48	72	96					
Yolk sac edema	+	+	+	+	♦		♦♦	♦	♦
Pericardial edema	+	+	+	+	♦♦	♦	♦♦	♦	♦♦
Blood accumulation in the yolk sac region	+	+	+	+	♦♦	♦♦		♦	♦♦
Blood accumulation in the brain region	+	+	+	+		♦		♦	♦
Underdeveloped head region	+	+	+	+				♦	
Scoliosis		+	+	+	♦	♦	♦	♦	♦
Blood accumulation in the tail region	+	+	+	+				♦	
Underdeveloped tail region	+	+	+	+	♦♦	♦♦			
Necrosis of the apical part of the tail			+	+		♦			
Growth retardation	+	+	+	+		♦♦			
Delay or anomaly in the absorption of the yolk sac		+	+	+	♦	♦	♦	♦	♦♦
Non-hatched			+	+	♦♦	♦♦	♦	♦♦	♦♦
Delay/absence of pigmentation formation		+	+	+	♦	♦♦	♦	♦	♦

♦ recorded sublethal endpoint; ♦♦ most frequently recorded sublethal endpoint

Table S2. Table of the p-value results of the one-way ANOVA test for multiple comparisons between the tested samples (GUA, CAT, and their nitrified intermediates), representing their impact on sensorial [a) eye area], skeletal [a) head height], and physiological parameters [c) yolk and d) pericardial sac area].

a) Eye area

	**C**	**CAT**	**4NC**	**GUA**	**5NG**
**CAT**	0.2275				
**4NC**	< 0.0001	0.0950			
**GUA**	< 0.0001	0.0003	0.0825		
**5NG**	0.0211	0.9837	0.9565	0.0118	
**4,6DNG**	0.5602	> 0.9999	0.0665	0.0002	0.9176

b) Head height

	**C**	**CAT**	**4NC**	**GUA**	**5NG**
**CAT**	0.2094				
**4NC**	0.0026	0.4097			
**GUA**	< 0.0001	< 0.0001	< 0.0001		
**5NG**	0.0011	0.1928	0.9908	< 0.0001	
**4,6DNG**	0.0022	0.2768	0.9972	< 0.0001	> 0.9999

c) Yolk sac area

	**C**	**CAT**	**4NC**	**GUA**	**5NG**
**CAT**	< 0.0001				
**4NC**	0.0285	0.0001			
**GUA**	< 0.0001	> 0.9999	0.0016		
**5NG**	0.0300	0.0010	0.9999	0.0057	
**4,6DNG**	0.0260	0.0049	0.9961	0.0171	0.9999

d) Pericardial sac area

	**C**	**CAT**	**4NC**	**GUA**	**5NG**
**CAT**	< 0.0001				
**4NC**	0.9986	< 0.0001			
**GUA**	0.0813	0.0617	0.1686		
**5NG**	0.7968	0.0004	0.9449	0.6337	
**4,6DNG**	0.6389	0.0067	0.8292	0.9095	0.9985

```
zfCA     1 MAHAWGYGPADGPESWAESFPIANGPRQSPIDIVPTQAQHDPSLKHLKLKYDPATTKSIL
hCA2     1 MSHHWGYGKHNGPEHWHKDFPIAKGERQSPVDIDTHTAKYDPSLKPLSVSYDQATSLRIL

zfCA    61 NNGHSFQVDFVDDDNSSTLAGGPITGIYRLRQFHFHWGSSDDKGSEHTIAGTKFPCELHL
hCA2    61 NNGHAFNVEFDDSQDKAVLKGGPLDGTYRLIQFHFHWGSLDGQGSEHTVDKKKYAAELHL

zfCA   121 VHWNTKYPNFGEAASKPDGLAVVGVFLKIGAANPRLQKVLDALDDIKSKGRQTTFANFDP
hCA2   121 VHWNTKYGDFGKAVQQPDGLAVLGIFLKVGSAKPGLQKVVDVLDSIKTKGKSADFTNFDP

zfCA   181 KTLLPASLDYWTYEGSLTTPPLLESVTWIVLKEPISVSPAQMAKFRSLLFSSEGETPCCM
hCA2   181 RGLLPESLDYWTYPGSLTTPPLLECVTWIVLKEPISVSSEQVLKFRKLNFNGEGEPEELM

zfCA   241 VDNYRPPQPLKGRKVRASFK
hCA2   241 VDNWRPAQPLKNRQIKASFK
```

Figure S1. Comparison of zebrafish carbonic anhydrase CAH-Z (UniProtKB Q92051) and human carbonic anhydrase 2 (UniProtKB P00918) protein sequences. Fasta sequences are taken from UniProt. Multiple sequence alignment was generated using T-Coffee and the output of T-Coffee was formated using Boxshade. Identity (165 out of 260 amino acids are identical, 63%) and positives (altogether 198 out of 260 amino acids are positive substitutions, 76%) were calculated by Protein BLAST.
